# Advances in Traditional Chinese Medicine for chronic obstructive pulmonary disease through multi-omics approaches

**DOI:** 10.3389/fcell.2026.1761374

**Published:** 2026-01-28

**Authors:** Kun Yang, Jun Wang, Yizhao Ma, Hailong Zhang

**Affiliations:** 1 Department of Respiratory Diseases, The First Affiliated Hospital of Henan University of Chinese Medicine, Zhengzhou, China; 2 The First Clinical Medical School, Henan University of Chinese Medicine, Zhengzhou, China; 3 Collaborative Innovation Center for Chinese Medicine and Respiratory Diseases Co-constructed by Henan Province and Ministry of Education of P.R. China, Henan University of Chinese Medicine, Zhengzhou, China

**Keywords:** biomarkers, chronic obstructive pulmonary disease, lung-gut axis, multi-omics, precision Traditional Chinese Medicine, systems biology, Traditional Chinese Medicine

## Abstract

Chronic obstructive pulmonary disease (COPD) is a common and complex heterogeneous chronic inflammatory airway disorder characterized by multifactorial pathogenesis and limited therapeutic options. It remains one of the leading causes of morbidity and mortality worldwide, severely impairing patients’ quality of life and life expectancy. Traditional Chinese Medicine (TCM), with its holistic perspective, syndrome differentiation, and multi-component formulations, is widely used in the adjunctive treatment and rehabilitation of COPD. Evidence suggests that TCM can relieve symptoms, reduce acute exacerbations, and enhance quality of life. However, the “multi-component-multi-target-multi-pathway” nature of TCM formulas has long made their mechanisms difficult to systematically elucidate. In recent years, the rapid development of high-throughput multi-omics technologies has provided unprecedented opportunities to decipher the systems biology mechanisms underlying TCM treatment of COPD. Multi-dimensional data from network pharmacology, genomics, transcriptomics, proteomics, metabolomics, and microbiomics can comprehensively reveal disease-related molecular changes. These findings provide scientific evidence for the objectification of TCM syndromes, the identification of biomarkers, and the clarification of multi-target mechanisms in key herbal formulas, such as Bufei Jianpi granules. Moreover, multi-omics studies have also promoted exploration of emerging fields such as the “lung-gut axis”, providing new theoretical perspectives for understanding the complex pathological processes of COPD. This review systematically summarizes recent advances in TCM-based prevention and treatment of COPD using multi-omics strategies. Key progress includes the application of network pharmacology and pharmacogenomics in component-target prediction, the role of transcriptomics/proteomics in molecular target validation, and the value of metabolomics and microbiomics in uncovering metabolic reprogramming and lung-gut axis regulation. Integrated multi-omics approaches also demonstrate significant potential in biomarker discovery and the development of precision TCM. In addition, this review provides a critical evaluation of current trends, potential breakthroughs, challenges, and opportunities in advancing TCM from empirical medicine toward evidence-based and precision medicine, aiming to offer systematic and comprehensive theoretical foundations and research perspectives for TCM-based COPD therapy.

## 1 Introduction

Chronic obstructive pulmonary disease (COPD) is a common progressive inflammatory lung disorder with high morbidity and mortality, imposing a substantial social and economic burden (Jiao et al., 2022). Its pathological hallmarks include persistent respiratory symptoms, airflow limitation, chronic inflammatory dysregulation, and emphysematous destruction (López-Campos et al., 2020; Yoshida and Tuder, 2007). COPD typically arises from airway and/or alveolar abnormalities induced by exposure to harmful particles and gases, such as cigarette smoke. The pathogenesis is primarily associated with chronic airway and pulmonary inflammation, oxidative stress, protease/antiprotease imbalance, and apoptosis (Van Eeden and Sin, 2013; Wang et al., 2017). As the disease progresses, cumulative pathological changes in the lungs significantly impair respiratory function. More critically, COPD is often accompanied by prominent extrapulmonary comorbidities, including musculoskeletal dysfunction, cardiovascular diseases, and gastrointestinal disorders (Keely et al., 2012; Rabe and Watz, 2017), which pose major challenges for its management.

Current therapeutic options for COPD remain limited, with pharmacological interventions offering only symptomatic relief. High-dose inhaled corticosteroids are widely used, but their efficacy is largely confined to reducing the frequency of acute exacerbations. When combined with bronchodilators, they may provide additional symptomatic improvement (Yang et al., 2012). However, many patients exhibit poor responsiveness to corticosteroid therapy (Jones et al., 2017), and such treatments do not address the underlying drivers of disease progression, reverse tissue damage, or reduce mortality. Moreover, they increase the risk of severe respiratory infections and pneumonia (Calverley et al., 2007). In contrast, Traditional Chinese Medicine (TCM), characterized by holistic regulation, syndrome differentiation, and synergistic multi-component formulations, has demonstrated considerable potential in alleviating COPD symptoms (Fan et al., 2020; Zhao et al., 2017). According to TCM theory, COPD patients often present with lung-kidney deficiency syndromes. Therefore, reinforcing the lung, strengthening the spleen, and tonifying the kidney constitute a fundamental therapeutic principle in TCM-based treatment of COPD (Chen et al., 2014; Liao et al., 2017). Clinical studies suggest that TCM interventions can improve respiratory symptoms, reduce acute exacerbations, and enhance quality of life. For instance, Yupingfeng granules have been shown to alleviate acute exacerbations in stage II-III COPD patients and improve conditions in stage II-IV patients (Ma et al., 2018). Bufei Yishen granules can restore Th17/Treg balance by regulating STAT3 and STAT5 activation, thereby ameliorating COPD symptoms (Zhao et al., 2018a). Liuweibuqi capsules improve COPD symptoms through modulation of JAK1/STAT3 signaling (Wang et al., 2017). However, the multi-component and multi-target nature of TCM formulations poses significant challenges for mechanistic elucidation using conventional single-target or reductionist research approaches.

The rapid development of omics technologies, including transcriptomics, proteomics, metabolomics, and microbiomics, has provided unprecedented opportunities to systematically investigate disease mechanisms across multiple biological layers (Gao et al., 2024; Cichonska et al., 2015). More importantly, unlike conventional single-omics approaches, multi-omics integration enables the identification of coordinated molecular networks rather than isolated biomarkers, making it particularly suitable for deciphering the complex therapeutic mechanisms of TCM. In recent years, multi-omics approaches have increasingly been applied to COPD research, facilitating mechanistic interpretation of TCM interventions at the systems level (Jiashuo et al., 2022).

In this review, we provide a comprehensive and critical synthesis of multi-omics applications in TCM-based COPD research, with a particular focus on how these approaches elucidate multi-target synergistic mechanisms, lung–gut axis regulation, and syndrome-specific molecular signatures. By systematically bridging classical TCM concepts with modern systems biology frameworks, this review offers a unique perspective that extends beyond descriptive summaries and highlights emerging directions for mechanism-oriented and translational TCM research, further underlying the therapeutic advantages of classical herbal formulas.

## 2 Pathological mechanisms and clinical efficacy of TCM in COPD

According to fundamental TCM theory, COPD is closely linked to dysfunction of the lung, spleen, and kidney. This association has been validated by multiple animal experiments and clinical observations. For instance, Wang C et al. (2015) established COPD rat models with lung deficiency syndrome (Fei Xu Zheng) by cigarette smoke exposure combined with intratracheal lipopolysaccharide instillation. Treatment with Liuweibuqi capsules modulated the JAK1/STAT3 pathway and MMP9/TIMP1 expression, leading to improvements in symptoms, lung function, and pulmonary histopathology. These findings suggest that TCM may alleviate COPD symptoms by regulating relevant signaling pathways. Another study involving 120 rats (Yange et al., 2015) developed stable COPD models through repeated smoke exposure and bacterial infection. Rats were treated with different herbal formulations, including Bufei Jianpi, Bufei Yishen, and Yiqi Zishen. It was found that these treatments enhanced pulmonary function indicators such as tidal volume and peak expiratory flow, while also increasing femoral weight, bone density, and bone mineral content. These results indicate that TCM has beneficial preventive and therapeutic effects on COPD complicated with osteoporosis.

Moreover, studies on the pathological mechanisms of TCM in COPD have demonstrated that inflammatory responses, oxidative stress, and apoptosis are critical contributors to disease progression. Several herbal medicines exert therapeutic effects by modulating these pathological processes. For instance, Bufei Yishen improved lung function and attenuated pathological changes in COPD rats by regulating pathways related to unsaturated fatty acid, phenylalanine, and phospholipid metabolism (Yang et al., 2015). In terms of oxidative stress, Bufei Yishen granules combined with acupoint application alleviated inflammatory responses in COPD rats by modulating the peroxisome proliferator-activated receptor-γ (PPARγ) signaling pathway, thereby suppressing oxidative stress (Li et al., 2016). In the study, superoxide dismutase (SOD) activity in serum and bronchoalveolar lavage fluid was significantly reduced, and malondialdehyde (MDA) levels were increased in the Model group. However, treatment elevated SOD activity, decreased MDA levels, and enhanced PPARγ mRNA and protein expression. These findings indicate that the combined therapy mitigates oxidative stress by activating the PPARγ signaling pathway. Inflammation is another key pathological process in COPD. Zhou et al. (2016) reported that Liujunzi Tang attenuated inflammatory responses in cigarette smoke-induced COPD model mice by inhibiting nuclear factor-κB activation and reducing the production of inflammatory cytokines, including tumor necrosis factor-α (TNF-α), interleukin-1β (IL-1β), and IL-6. Similarly, Yu-Ping-Feng-San suppressed the transforming growth factor-β1 (TGF-β1)/Smad2 signaling pathway, thereby decreasing inflammatory cell infiltration and collagen deposition in the lung tissues of COPD rats, while lowering IL-1β, IL-6, and TNF-α levels (Yang et al., 2016). Collectively, these studies reveal the pathological mechanisms of TCM in COPD from different perspectives and provide theoretical support for its clinical application.

**FIGURE 1:**
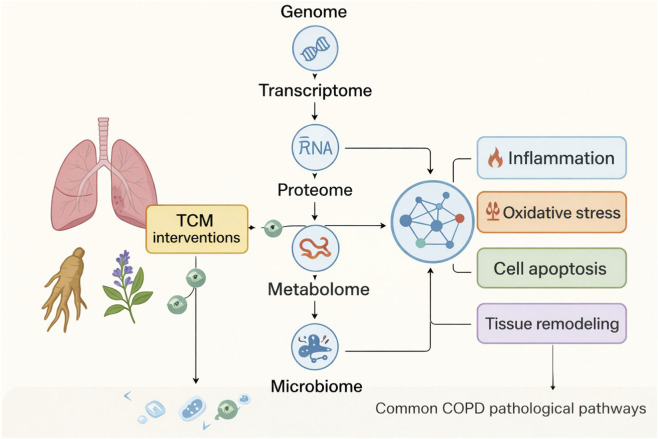
Multi-omics approaches reveal the molecular mechanisms of TCM in the treatment of COPD.

From a clinical perspective, a study of 300 patients with stable COPD using TCM syndrome classification Wang H. et al. (2015) found that as the disease progressed, syndromes evolved from single-organ to multi-organ involvement, accompanied by gradual increases in the BODE index and CAT scores. This suggests that TCM syndromes are closely related to COPD severity. Besides, Wang et al. (Haifeng et al., 2015) conducted a systematic review and meta-analysis of 37 randomized clinical trials involving 3,212 patients. Compared with conventional therapy alone, TCM combined with conventional treatment improved forced expiratory volume in one second (MD 0.12 L, 95% CI 0.08–0.16), reduced acute exacerbations (OR -0.86, 95% CI -1.13 to −0.60), enhanced quality of life (MD -4.36, 95% CI -7.12 to −1.59), and increased exercise tolerance (MD 36.66 m, 95% CI 24.57–48.74). These findings further confirm the efficacy of TCM in stable COPD. However, clinical trials show heterogeneity in sample size, blinding, and follow-up duration, and outcome assessment lacks standardized biological markers. Therefore, integrating multi-omics biomarkers may provide more definitive evidence for the clinical application of TCM in COPD (Figure 1).

## 3 Network pharmacology and pharmacogenomics: predicting the mechanisms of TCM in COPD

Network pharmacology, an emerging discipline integrating systems biology and multi-omics technologies, provides new perspectives for investigating the mechanisms of TCM formulas (Wu and Wu, 2015). By integrating herbal compound databases, drug target prediction, and disease-related gene networks, it enables the construction of “compound-target-pathway” networks. These networks help to systematically elucidate the synergistic mechanisms underlying the multi-component, multi-target, and multi-pathway actions of TCM formulas (Yu et al., 2012). In studying the therapeutic effects of TCM formulas, network pharmacology allows comprehensive analysis of interactions between active compounds and disease-related targets. Potential targets of chemical probes can be identified through compound-target interaction networks, protein-protein interaction (PPI) networks, and pathway enrichment analyses [e.g., Kyoto Encyclopedia of Genes and Genomes (KEGG), Gene Ontology (GO)]. Such findings provide clues for drug repurposing and novel drug development (Cichonska et al., 2015). Moreover, combined with molecular docking techniques, the binding activity between active compounds and targets can be further validated, offering stronger evidence for elucidating the mechanisms of TCM formulas.

Network pharmacology has been extensively applied in COPD research and provides a valuable approach to elucidating the mechanisms of TCM in COPD treatment. Multiple studies have shown that herbal medicines exert therapeutic effects by modulating signaling pathways related to inflammation, immunity, and oxidative stress. For example, Yin et al. (2020) identified 23 active compounds and 83 COPD-related target genes of Yupingfeng granules through network pharmacology analysis. These genes were mainly linked to glucocorticoid and steroid hormone responses, while apoptosis and the HIF-1 signaling pathway played central roles in its therapeutic effects. In another study, Rheum palmatum (Chinese rhubarb) was found to contain eight active compounds and 90 related targets (Xue and Shi, 2021). These targets were primarily involved in apoptosis regulation, inflammatory responses, and associated signaling pathways. Molecular docking further revealed different binding affinities between active compounds and key targets, suggesting that Rheum palmatum may exert therapeutic effects by modulating these targets. Additionally, research on Qiwei Putao powder, integrating network pharmacology with experimental validation, demonstrated that its active components (isoliquiritigenin, pterostilbene, and quercetin) could activate nuclear factor erythroid 2-related factor 2 (Nrf2), enhance cellular antioxidant responses, and alleviate pathological changes in COPD mice. These findings provide theoretical support for its clinical application (Deng et al., 2021).

Bufei Yishen as a representative example (Wu et al., 2020), 30 active compounds and 37 core targets were identified by network pharmacology and molecular docking. These compounds and targets were primarily involved in regulating biological functions such as responses to biological and chemical stimuli, diverse cellular processes, immunity, and metabolism. Several signaling pathways were found to play critical roles in its therapeutic effects, including IL-17, Toll-like receptor (TLR), TNF, and HIF-1. Furthermore, Zhou et al. (Cichonska et al., 2015) constructed compound-target and PPI networks using TCM systems pharmacology. Their analysis revealed that Pericarpium Citri Reticulatae (Chenpi) may exert therapeutic effects by modulating key active compounds (e.g., nobiletin, naringenin, hesperetin) and core targets (e.g., AKT1, TP53, IL6, VEGFA, MMP9). These interactions are involved in the PI3K-Akt and MAPK signaling pathways, thereby preventing COPD progression and its transition to lung cancer. Collectively, these studies highlight the capacity of network pharmacology to uncover the potential mechanisms of TCM in COPD and to provide new strategies and therapeutic targets for its treatment.

Although pharmacogenomics and network pharmacology have made certain progress in TCM research, they still face multiple limitations. For instance, the coverage and accuracy of database annotations remain inconsistent. Static networks cannot capture spatiotemporal dynamics. Integrated consideration of absorption, distribution, metabolism, and excretion (ADME) and pharmacokinetics is lacking. In addition, constructed network models often overlook factors such as cellular microenvironment and genetic background, leading to discrepancies between predicted results and actual conditions (Cichonska et al., 2015). Therefore, network pharmacology should be closely integrated with multi-omics data and experimental validation to enhance the biological reliability and clinical translational potential of drug-target interaction predictions.

## 4 Advances in genomics and metagenomics in TCM interventions for COPD

With the rapid development of high-throughput sequencing technologies, genomics (including host genomics) and metagenomics have become increasingly valuable in investigating TCM interventions for COPD. Unlike traditional single-molecule indicators, multi-omics strategies based on whole-genome and metagenomic sequencing (including bacterial, viral, and fungal omics) can identify key molecular networks associated with TCM syndromes and formula responses within the “host-microbe-metabolism” interaction network. These strategies provide new technical routes for mechanistic research of TCM, the discovery of syndrome-related biomarkers, and the construction of precision herbal prescriptions.

In host genomics (particularly genetic polymorphisms/pharmacogenomics), high-throughput sequencing such as whole-genome sequencing (WGS) or whole-exome sequencing (WES) can be used to identify genetic variations related to the metabolism, responsiveness, and safety of herbal compounds. For example, a recent study employed deep metagenomic sequencing to simultaneously perform host genotyping (SNP detection) and respiratory microbiome profiling. More than 5 million SNPs were identified in sputum samples from COPD patients, and host genetic variations (such as specific SNPs) were found to be significantly associated with particular microbial species and functional modules. This study demonstrated that host genetics is an important factor impacting airway microecology and revealed that gene-microbe interactions may be involved in COPD pathogenesis (Gao et al., 2024). With the advancement of deep metagenomics, researchers have begun to focus on the role of pulmonary and gut viromes in COPD onset and progression. Liu et al. (2024) combined deep metagenomic sequencing with bacterial 16S rRNA sequencing and showed that gut virome diversity was associated with COPD severity and exacerbations. Moreover, complex exchanges were observed between lung and oropharyngeal microbiota, providing new directions for investigating how TCM may affect viral/phage ecology, inflammation, and COPD treatment.

Furthermore, integrating metagenomic data with metabolomic profiles has provided direct evidence for how TCM impacts the host through the microecology-metabolism network (Bowerman et al., 2020). By associating metagenomic functional annotations (e.g., microbial metabolic pathways) with host-derived small-molecule metabolites (measured in serum and urine via metabolomics), Di et al. (2025) demonstrated that Qi-Huo-Yi-Fei formula significantly modulates microbial metabolic products such as short-chain fatty acids, indole metabolites, and bile acids. These alterations regulate metabolism and gut microbiota, effectively improving lung function and suppressing COPD progression. Such integrative analyses also support the “lung-gut axis” mechanism, in which TCM regulates gut microbiota and their metabolites to affect host immunity and inflammatory responses (Cheng and Zhang, 2024), offering a promising strategy for COPD treatment. However, current studies are largely confined to animal models, lack clinical validation, and have small sample sizes (e.g., n < 100). Future prospective clinical cohorts with multi-timepoint dynamic monitoring are needed to enhance reliability.

## 5 Transcriptomics in elucidating the molecular mechanisms and research progress of TCM interventions for COPD

Transcriptomics enables comprehensive analysis of gene expression profiles in COPD patients, thereby revealing the molecular mechanisms underlying disease onset and progression. Studies have shown that peripheral blood gene expression modules in COPD patients are closely associated with disease phenotypes (Reinhold et al., 2017). Through gene co-expression network analysis of the COPDGene, ECLIPSE, and TESRA cohorts, Reinhold et al. identified several gene modules related to airflow obstruction and emphysema. These modules were enriched in functional categories such as immune and defense responses and contained cell type-specific genes from natural killer cells, dendritic cells, and neutrophils. Together, these findings provide new insights into COPD pathogenesis. In cigarette smoke-induced COPD mouse models, Li et al. (2017) demonstrated via transcriptomic analysis that taurine ameliorated particulate matter-induced emphysema by restoring the expression of mitochondrial NADH dehydrogenase genes. Furthermore, Farahani et al. (2017) found, through transcriptomic analysis, that cytokine gene expression related to Th17/Treg cells was imbalanced in peripheral blood mononuclear cells of COPD patients and correlated with disease severity. Collectively, these studies underscore the value of transcriptomics in uncovering gene expression changes during COPD pathogenesis and highlight its potential to identify novel therapeutic targets.

Transcriptomics also provides a powerful tool for exploring the molecular mechanisms of TCM in COPD treatment. For example, in the study of Tong Sai granules for acute exacerbation of COPD (AECOPD) (Tao et al., 2023), Liu et al. combined transcriptomic analysis with experimental validation in AECOPD rat models and airway epithelial cells. Their findings showed that Tong Sai granules alleviated AECOPD symptoms by modulating the MAPK-SIRT1-NF-κB signaling pathway to inhibit cellular senescence. In another study, Yang et al. (2024) examined Qing-Jin-Hua-Tan decoction for COPD by integrating *in vitro* cell experiments and *in vivo* animal studies with network pharmacology, transcriptomics, and metabolomics. They discovered that the active compound acacetin exhibited strong binding affinity to key target proteins and exerted therapeutic effects by inhibiting inflammatory signaling (e.g., IL-1β, IL-6, TNF, IκB-NF-κB, TLR, and MAPK) and apoptosis, while regulating sphingolipid and choline metabolism. Li et al. (2014) further validated the systemic effects of Bufei Yishen at the transcriptomic level (see Section 3 for an overview of its network pharmacology). Gene expression profiling in COPD rat lung tissues revealed that Bufei Yishen modulated transcripts associated with mitochondrial electron transport, inflammatory signaling, and cytoskeletal organization, providing downstream molecular evidence supporting its predicted multi-target regulatory network. Whereas Cao et al. (2023) highlighted mitochondrial electron transport and energy metabolism as central regulatory nodes based on integrated transcriptomic, proteomic, metabolomic, and systems pharmacology analyses, enabled identification of upstream mitochondrial and energy metabolism remodeling. Despite these apparent differences, both studies employed comparable COPD rat models and overlapping tissue types, suggesting that the divergence arises primarily from differences in analytical depth rather than biological inconsistency. Importantly, mitochondrial dysfunction, lipid metabolic imbalance, and oxidative stress are intrinsically interconnected processes in COPD pathogenesis. We therefore propose that Bufei Yishen exerts therapeutic effects through a hierarchical regulatory cascade, in which modulation of mitochondrial energy metabolism leads to downstream normalization of lipid metabolism, redox homeostasis, and inflammatory signaling. Since transcriptomics data may be affected by sample heterogeneity and batch effects, and cannot directly reflect protein function, this integrative interpretation reconciles previously reported findings and underscores the necessity of multi-omics approaches for elucidating the systemic mechanisms of complex TCM formulas.

## 6 Proteomics in elucidating the molecular mechanisms and research progress of TCM interventions for COPD

Proteomics, as a methodology capable of directly reflecting cellular functional states and signaling pathway activity, plays an important role in uncovering the mechanisms of TCM in COPD. Compared with genomics or transcriptomics, proteomics provides a more accurate representation of cellular functions, pathway activity, and dynamic changes in post-translational modifications (PTMs). This offers unique advantages in revealing the “multi-component-multi-target-multi-pathway” effects of TCM (Giudice and Petsalaki, 2017). In recent years, approaches ranging from global protein expression profiling to detailed phosphoproteomics have generated abundant experimental evidence. These studies have helped clarify how TCM regulates critical pathological processes such as inflammatory responses, oxidative stress, extracellular matrix remodeling, and immune homeostasis (Li X. et al., 2015). Using high-resolution mass spectrometry (LC-MS/MS), researchers can comprehensively quantify protein expression in lung tissues, bronchoalveolar lavage fluid (BALF), or serum/plasma. Combined with labeling methods (e.g., TMT/iTRAQ) or label-free approaches, differential expression proteins (DEPs) can be identified, and bioinformatics analyses (GO, KEGG, PPI networks) can be employed to elucidate the core biological processes modulated by TCM.

Studies have shown that certain TCMs exert therapeutic effects on COPD by modulating the expression of key proteins. For example, in a study on Chuanbei Pipa dripping pills Ma et al. (2019), performed phosphoproteomic analysis on smoke-exposed early-stage COPD model mice. They combined molecular biology methods to examine the effects of its major compounds on signaling pathways related to inflammation, cellular contraction, and fibrosis. The results indicated that the active component peimine acted on the epidermal growth factor receptor (EGFR). By inhibiting EGFR signaling, peimine improved lung function, reduced pulmonary fibrosis, and prevented COPD exacerbation. Zhang et al. (2023) employed proteomic and metabolomic analyses to investigate astragaloside IV (AST). They found that AST exerts protective effects against COPD-induced lung injury and fibrosis by targeting the GTP-GDP domain of RAS. It downregulates the RAS/RAF/FoxO signaling pathway, inhibits fibroblast-to-myofibroblast differentiation, and reduces the expression of inflammatory factors and extracellular matrix. These actions further attenuate epithelial-mesenchymal transition, thereby contributing to its protective role in COPD. These studies, through proteomic analysis, have clarified the key protein targets and signaling pathways involved in TCM treatment of COPD, providing important evidence for mechanistic research. Integrating transcriptomics with proteomics allows a more comprehensive understanding of the molecular mechanisms underlying TCM interventions in COPD. For example, in the case of Tong Sai granules (Tirelli et al., 2024), transcriptomic analysis revealed their regulatory effects on MAPK-SIRT1-NF-κB pathway-related gene expression. Whereas, proteomic methods confirmed corresponding changes in protein expression. Cell and animal experiments further demonstrated that Tong Sai granules reduced the expression levels of proteins such as p-p38, p-ERK1/2, p-JNK, and p-p65, while increasing SIRT1 protein expression. These findings confirmed their mechanism of inhibiting cellular senescence and alleviating AECOPD symptoms through modulation of this signaling pathway (Table 1). However, proteomics coverage is limited by technical constraints, and the dynamic range is narrow; future high-depth sequencing and phosphoproteomics are needed for supplementation.

**TABLE 1:** Transcriptomic and proteomic studies of TCM in COPD prevention and treatment: validation of molecular targets and functional mechanisms.

TCM	Subjects/Models	Sample type	Omics approach	Main mechanisms and key findings	References
Qing-Jin-Hua-Tan decoction	*In vitro* COPD cell model	BEAS-2B cells	Transcriptomics + Metabolomics + network pharmacology	Regulates sphingolipid (SL) metabolism directed by inflammatory signaling and apoptosis	Yang et al. (2024)
Bufei Yishen	COPD rat model	Lung tissues	Transcriptomics (gene chip)	Modulates interleukin production, myosin filament assembly components, and mitochondrial electron transport-related molecules to improve COPD	Li J et al. (2014)
Bufei Yishen	COPD rat model	Lung tissues	Integrated transcriptomics + proteomics + metabolomics + pharmacology	Regulates 195 differential proteins involved in lipid metabolism, oxidative stress, inflammatory responses, and cell junction pathways	Zhao P et al. (2018)
Bufei Jianpi formula	COPD rat model	Lung tissues	Integrated transcriptomics + proteomics + phosphoproteomics + metabolomics + systems pharmacology	Systematically modulates lipid metabolism, inflammatory signaling pathways, oxidative stress, and focal adhesion	Zhao P et al. (2017)
Tong Sai granules	AECOPD rat model	Lung tissues/bronchial epithelial cells	Transcriptomics + MS and interaction network analysis	Regulates MAPK-SIRT1-NF-κB pathway and alleviates cellular senescence to improve AECOPD	Tao et al. (2023)
Chuanbei Pipa dripping pills	COPD mouse model	Lung tissues	Phosphoproteomics	Peiminine targets EGFR and modulates downstream inflammation- and fibrosis-related pathways; improves pulmonary fibrosis and reduces risk of COPD exacerbation	Ma X et al. (2019)
Astragaloside IV (AST)	COPD mouse model	Lung tissues	Proteomics + metabolomics	Targets the GTP-GDP domain of RAS and downregulates the RAS/RAF/FoxO signaling pathway, thereby protecting lungs against COPD-induced injury and fibrosis	Zhang M et al. (2023)

## 7 Metabolomics and microbiomics: revealing metabolic reprogramming and the lung-gut axis in TCM interventions for COPD

Metabolomics and microbiomics represent critical downstream layers in multi-omics investigations of COPD, providing direct readouts of systemic metabolic states and host–microbe interactions. Unlike upstream genomic, transcriptomic, or proteomic alterations, which primarily reflect regulatory potential, metabolomic and microbiomic profiles capture functional outcomes of disease progression and therapeutic intervention. In the context of TCM, these omics layers offer a quantitative framework to reinterpret classical concepts such as “phlegm accumulation,” “heat toxicity,” and “deficiency syndromes” as measurable metabolic and microecological disturbances.

Metabolomics employs gas or liquid chromatography coupled with mass spectrometry (GC-MS, LC-MS/MS) and nuclear magnetic resonance (NMR) to directly measure small-molecule metabolites, including lipids, amino acids, bile acids, and short-chain fatty acids. These metabolites directly reflect alterations in energy metabolism, redox balance, and inflammatory signaling—core pathological processes in COPD. Microbiomics, encompassing 16S rRNA gene sequencing, shotgun metagenomics, and metatranscriptomics, enables comprehensive characterization of gut and pulmonary microbial composition and function. Integration of metabolomic and microbiomic data allows the construction of microbe–metabolite–host interaction networks, providing molecular evidence for the lung–gut axis as a key regulatory route in COPD pathophysiology and TCM intervention. Accumulating evidence indicates that COPD is accompanied by profound metabolic reprogramming and microbial dysbiosis, including impaired lipid and amino acid metabolism, mitochondrial dysfunction, and altered gut microbial composition. TCM interventions can partially reverse these disturbances, thereby restoring systemic homeostasis. Importantly, these metabolomic and microbiomic changes are increasingly recognized as downstream manifestations of upstream regulatory events, such as transcriptomic reprogramming and proteomic remodeling of mitochondrial, inflammatory, and oxidative stress pathways (see Sections 5, 6), rather than independent or isolated mechanisms (Wu et al., 2023; Zhang et al., 2024; Cao et al., 2023).

Clinical metabolomics studies have begun to provide direct molecular evidence linking TCM syndrome differentiation with metabolic regulation. A representative study published in 2024 investigated the molecular effects of TCM interventions in patients with acute exacerbation of COPD (AECOPD) (n = 69). In this study (Zhang et al., 2024), Patients with phlegm-heat obstructing the lung (PH, n = 41) and phlegm-dampness obstructing the lung (PD, n = 28) received standardized Chinese herbal medicine granules (Qingre Huatan or Zaoshi Huatan granules) in addition to conventional therapy for a 7 day intervention period. Untargeted serum metabolomics identified distinct sets of differential metabolites in each syndrome group. In phlegm-heat patients, several lysophosphatidylcholines were negatively correlated with inflammatory markers and symptom scores, whereas in phlegm-dampness patients, metabolites related to amino acid and carnitine metabolism showed positive correlations with disease severity indicators. These findings demonstrate that metabolomic signatures can serve as objective biomarkers bridging traditional syndrome classification and modern clinical evaluation, particularly during acute inflammatory phases of COPD.

From a stage-specific perspective, omics profiles differ markedly between AECOPD and stable COPD. During acute exacerbations, metabolomic and proteomic signatures are dominated by inflammatory activation and oxidative stress, with perturbations in arachidonic acid metabolism and cytokine-associated pathways. Correspondingly, TCM strategies emphasize “clearing heat and resolving phlegm” to suppress excessive inflammation and mitigate acute tissue injury. In contrast, stable COPD is characterized by chronic immune–metabolic imbalance, including impaired energy metabolism, mitochondrial dysfunction, and persistent gut microbiota dysbiosis. Although large-scale randomized multi-omics clinical studies in stable COPD remain limited, existing clinical observations and animal data suggest that TCM formulas targeting “tonifying deficiency” can modulate lipid metabolism, amino acid biosynthesis, and host–microbiota interactions, thereby supporting long-term disease management.

Microbiomic studies further reinforce the role of the lung–gut axis in COPD. COPD patients frequently exhibit an altered Firmicutes-to-Bacteroidetes ratio and reduced abundance of beneficial short-chain fatty acid–producing bacteria. TCM interventions have been shown to partially restore microbial composition and functional capacity, increasing the abundance of genera such as Bifidobacterium, Faecalibacterium, and Blautia. These microbial shifts are closely correlated with improvements in metabolic profiles and inflammatory markers, suggesting that microbiota-derived metabolites act as key mediators linking gut ecology to pulmonary immune regulation. Cao et al. (2023) reported that TCM treatment in COPD patients with lung qi deficiency syndrome regulated metabolites related to energy and amino acid metabolism, thereby improving clinical outcomes. In terms of microbiomics, Lim et al. (2023) found that COPD patients exhibited an imbalance in the ratio of Firmicutes to Bacteroidetes in the gut microbiota, which could be corrected by TCM to restore intestinal microecology. Meanwhile, a related study (Di et al., 2025) analyzed serum and urine metabolomics in COPD mouse models. It was found that treatment with the Qi-Huo-Yi-Fei formula modulated 19 differential metabolites, involving pathways such as tyrosine metabolism, taurine and hypotaurine metabolism, the citric acid cycle, and tryptophan metabolism. In addition, the Qi-Huo-Yi-Fei formula increased the abundance of beneficial bacteria, including Bifidobacterium, Blautia, Faecalibacterium, and Parabacteroides. The differential metabolites were closely correlated with bacterial abundance and therapeutic outcomes, suggesting that the formula may exert its effects by regulating metabolism and gut microbiota. These findings indicate that changes in gut microbiota in COPD patients are closely associated with metabolic disorders and inflammatory responses. They also highlight the complex interactions between the microbiota and the host. In a study on Xuanbai Chengqi decoction for COPD patients with lung heat and intestinal excess syndrome (Jiao et al., 2022), metabolomic and microbiomic analyses revealed that disease progression was associated with imbalances in 41 differential metabolites in plasma, bronchoalveolar lavage fluid, and feces, as well as 82 bacterial taxa in the lung and gut. Treatment with Xuanbai Chengqi decoction reversed 30 differential metabolites and 65 bacterial changes.

Beyond descriptive associations, metabolomic and microbiomic alterations offer mechanistic insight into how TCM formulas exert synergistic effects. In complex formulas, primary regulation of host metabolic pathways may correspond to the dominant effects of “monarch” herbs, while microbiome modulation and secondary metabolic adjustments reflect the supportive roles of “minister” and “adjuvant” components. The coordinated regulation of metabolism, microbiota, and inflammation thus represents an omics-based manifestation of synergistic TCM interactions. Collectively, current evidence supports the view that TCM treats COPD through coordinated regulation of metabolic reprogramming and lung–gut axis homeostasis (Table 2).

**TABLE 2:** Metabolomics and microbiomics studies of TCM in COPD prevention and treatment: representative examples exploring the lung-gut axis.

TCM	Subjects/Models	Sample type	Omics approach	Key findings	References
Qi-Huo-Yi-Fei formula	COPD mice	Urine, serum	Metabolomics + gut microbiota analysis	Regulates the microbiota-metabolite axis to alleviate pulmonary inflammation	Di et al. (2025)
Xuanbai Chengqi decoction	Human/animal combined study (clinical samples + animal validation)	Plasma/BALF/feces	Microbiomics (16S/shotgun) + metabolomics	Reverses multiple differential metabolites and modulates lung and gut microbiota	Jiao J et al. (2022)
Bufei Yishen	COPD rats	Lung tissues, serum metabolites	Integrated transcriptomics, proteomics, metabolomics, and systems pharmacology	Modulates lipid metabolism, oxidative stress, cell junctions, and inflammatory pathways	Li J et al. (2016); Zhao P et al. (2018)
Qingre Huatan or Zaoshi Huatan granules	AECOPD patients	Serum metabolites	Metabolomics	Serum metabolites serve as potential objective indicators for evaluating TCM syndrome efficacy	Zhang et al. (2024)

## 8 Integration of multi-omics to elucidate the mechanisms and applications of TCM in COPD

With the rapid development of omics technologies and computational methods, multi-omics integration has emerged as a key strategy for elucidating the multi-target and multi-pathway synergistic effects of TCM formulas in COPD. Multi-omics integration generally refers to the combined analysis of data from different layers, including genomics/pharmacogenomics, transcriptomics, proteomics, metabolomics, and microbiomics. By applying approaches such as network biology, pathway enrichment, machine learning, and causal inference, researchers can construct cross-scale causal chains linking “components-targets-pathways-phenotypes”. This framework helps explain the holistic efficacy and individual variability of TCM from the molecular to cellular and even systemic levels, offering a comprehensive and systematic methodology for mechanistic research, identification of potential biomarkers, and evaluation of drug safety in TCM (Zhao et al., 2017; Zhao et al., 2018b).

In practice, this integration relies on a combination of network-based modeling, data fusion algorithms, and latent factor approaches. For example, weighted gene co-expression network analysis (WGCNA) is frequently used to identify gene or protein modules associated with inflammatory regulation or immune remodeling. Similarity network fusion (SNF) enables the integration of heterogeneous omics datasets by constructing patient- or sample-level similarity networks across different omics layers and merging them into a unified network representation. In addition, multi-omics factor analysis (MOFA) and related latent variable models extract shared and modality-specific factors, facilitating the identification of coordinated molecular signatures underlying COPD phenotypes and therapeutic responses.

**FIGURE 2:**
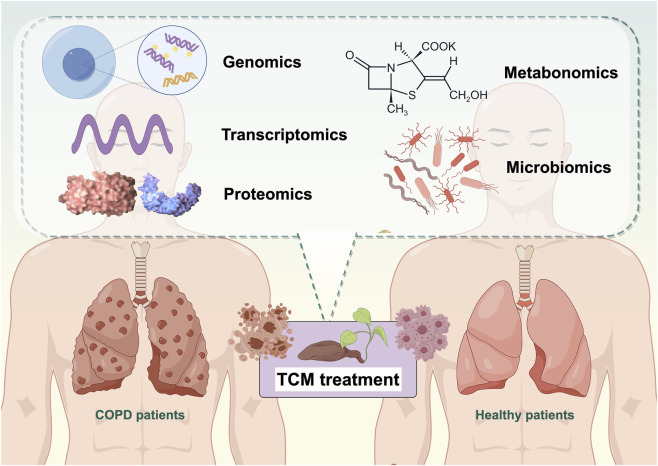
Multi-omics integration in elucidating the mechanisms of TCM for COPD treatment.

A representative integrative framework can be illustrated using Bufei Jianpi Formula as an example. Transcriptomic analyses may first identify immune-related gene modules associated with inflammatory suppression, while proteomics further reveals mitochondrial and oxidative phosphorylation alterations. Metabolomics can then link these molecular changes to lipid remodeling and redox balance, whereas microbiomics connects short-chain fatty acid–producing bacteria with systemic immune modulation. Integration strategies such as weighted gene co-expression network analysis and similarity network fusion enable the convergence of these heterogeneous datasets into coordinated molecular modules, providing a systems-level interpretation of TCM’s multi-target synergistic effects. Consistent with this framework, Wang et al. (2022) conducted a multi-omics analysis of Bufei Jianpi granules in COPD treatment. They found that the active components of the formula act on targets such as EGFR, ERK1, PAI-1, and p53. These targets regulate lung function, mucus secretion, inflammatory responses, and energy metabolism, ultimately improving COPD outcomes. Similar multi-omics integration strategies have been applied to Bufei-based formulas, including Bufei Yishen Formula (Heo et al., 2025), where transcriptomic, proteomic, metabolomic, and microbiomic data collectively reveal coordinated regulation of lipid metabolism, antioxidant pathways (e.g., Nrf2 signaling), inflammatory cascades, and gut microbiota dysbiosis (see Section 3–7 for details). This integration forms a complete “component-target-pathway-microbiota-metabolite-phenotype” chain, offering molecular and microecological evidence for the systemic efficacy of TCM formulas (Cao et al., 2023; Sauler et al., 2022) (Figure 2).

## 9 Future trends, challenges and prospects of TCM in COPD research

In summary, the application of multi-omics technologies has provided unprecedented opportunities to decode the complex, multi-component, and multi-target mechanisms underlying TCM interventions in COPD. By integrating diverse molecular layers, multi-omics approaches not only improve mechanistic interpretability but also enable a more objective evaluation of TCM efficacy, moving the field closer to precision medicine. This review uniquely emphasizes the convergence of omics-driven evidence with TCM holistic theory, highlighting how systems-level regulation, rather than isolated pathways, underlies the therapeutic advantages of classical herbal formulas. Numerous studies have confirmed that active compounds in TCM, including flavonoids, terpenoids, and phenolics, can exert therapeutic effects through multiple pathways. These include inhibiting inflammatory responses, alleviating oxidative stress, regulating apoptosis and autophagy, and improving airway remodeling (Schiller et al., 2019; Li et al., 2012). Such functional networks closely align with the core pathological processes of COPD, thereby laying a solid foundation for the further development of TCM in this domain. Multi-omics integration represents a critical advancement beyond parallel single-omics analyses. Computational approaches such as weighted gene co-expression network analysis, Bayesian network modeling, and similarity network fusion enable the identification of coordinated molecular modules underlying TCM interventions. However, current applications often lack standardized pipelines and rigorous validation.

Single-cell and spatial multi-omics technologies offer new opportunities for TCM research in COPD. To date, single-cell and epigenomic multi-omics studies specifically focusing on TCM interventions in COPD remain scarce. However, recent landmark studies, including the Human Lung Cell Atlas and subsequent COPD-focused scRNA-seq analyses, have demonstrated profound remodeling of epithelial, immune, and stromal cell populations in diseased lungs. For example, Sauler et al. identified disease-specific macrophage and epithelial cell states within the COPD alveolar niche, highlighting altered inflammatory signaling and impaired tissue repair capacity. Similarly, single-cell transcriptomic profiling of peripheral blood from COPD patients revealed systemic immune dysregulation, characterized by shifts in monocyte and T cell subpopulations. These findings provide a mechanistic cellular basis for COPD heterogeneity and suggest that TCM interventions may exert therapeutic effects by reprogramming specific pathogenic cell subsets, rather than globally suppressing inflammation. Importantly, single-cell omics offers a powerful framework for translating TCM syndrome concepts into quantifiable cellular phenotypes (Sauler et al., 2022; Heo et al., 2025). Such findings help clarify the mechanisms by which TCM modulates cellular functions and guide the development of “cell-targeted” intervention strategies. Spatial transcriptomics, in turn, preserve tissue architecture and cell–cell interactions, enabling interrogation of microenvironmental heterogeneity within the lung. In COPD, spatial transcriptomics has the potential to map inflammatory niches, epithelial–immune interfaces, and fibrotic remodeling zones that are obscured in dissociative assays. From a TCM perspective, spatial omics provides a unique opportunity to examine how herbal interventions modulate localized inflammatory microenvironments, rebalance immune cell infiltration, and restore epithelial barrier integrity. By linking spatial gene expression patterns to functional lung regions, spatial omics may bridge traditional holistic theories with modern tissue-level molecular evidence, offering unprecedented insight into how TCM regulates pulmonary heterogeneit (Schiller et al., 2019). For instance, examining the impact of TCM on gene expression in inflamed and normal lung areas of COPD patients can deepen understanding of therapeutic mechanisms. Epigenomic mechanisms, particularly histone modifications, represent a critical but underexplored dimension in COPD research and TCM studies. Unlike transient transcriptional changes, chromatin modifications can establish long-lasting regulatory states that influence immune memory, metabolic adaptation, and tissue remodeling—hallmarks of chronic COPD progression. Integrating TCM interventions into such single-cell frameworks with spatial tissue architecture and epigenomic regulation may enable the identification of cell-specific therapeutic responses and epigenetic regulation patterns that are not accessible through bulk omics approaches. This represents a critical future direction for advancing mechanism-driven and precision-oriented TCM research.

However, multi-omics integration faces challenges such as high data heterogeneity and lack of algorithmic standardization. For instance, the types, scales, and analytical methods of different omics datasets vary considerably, making effective integration an urgent issue to be addressed. In addition, the complex composition and diverse mechanisms of TCM formulas pose another challenge in accurately elucidating their multi-component and multi-target synergistic effects (Li et al., 2012). Also, insufficient clinical validation and lack of technical standardization will definitely affect the multi-omics-guided TCM application for COPD. Most transcriptomic and proteomic studies are exploratory in nature, relying on single animal models with limited sample sizes and lacking independent validation cohorts. In contrast, metabolomics studies often benefit from higher analytical reproducibility but are frequently cross-sectional and insufficiently powered for biomarker validation. Current clinical omics studies in COPD are largely constrained by small sample sizes, single-center designs, and short intervention durations. There is a notable absence of large-scale randomized controlled trials (RCTs) and real-world studies (RWS) integrating longitudinal multi-omics profiling with clinical outcomes. Future research should prioritize prospective cohorts that track the transition from stable COPD to AECOPD, enabling the identification of early warning omics biomarkers predictive of exacerbation risk. Furthermore, few studies apply rigorous statistical correction or cross-omics validation strategies. Future work requires the development of standardized pipelines (e.g., MOFA or SNF algorithms) to enhance the reliability and reproducibility of data integration. Meanwhile, clinical translation should be strengthened through prospective cohorts combined with multi-timepoint dynamic monitoring to validate the clinical utility of multi-omics biomarkers.

Nevertheless, with the advancement of multi-omics studies, an increasing number of biomarkers associated with TCM efficacy have been identified. These include specific metabolites, microbiota signatures, and protein pathway nodes. Integrative multi-omics strategies contribute to establishing a modern research paradigm for TCM in COPD (Li J. et al., 2015; Schüs et al., 2019). Emerging technologies, including single-cell sequencing, spatial transcriptomics, epigenomics, and even organoid models, will offer unprecedented opportunities to refine mechanistic understanding and translational relevance (Hasin et al., 2017). At the same time, the joint efforts of biology, medicine, pharmacy, and computer science with prospective clinical cohorts will be essential to fully realize the potential of multi-omics-guided TCM research for COPD, and ultimately provide more effective personalized therapeutic options for COPD patients (Duprey et al., 2020).
