# Correction: Advances in traditional Chinese medicine for chronic obstructive pulmonary disease through multi-omics approaches

**DOI:** 10.3389/fcell.2026.1799895

**Published:** 2026-02-10

**Authors:** Kun Yang, Jun Wang, Yizhao Ma, Hailong Zhang

**Affiliations:** 1 Department of Respiratory Diseases, The First Affiliated Hospital of Henan University of Chinese Medicine, Zhengzhou, China; 2 The First Clinical Medical School, Henan University of Chinese Medicine, Zhengzhou, China; 3 Collaborative Innovation Center for Chinese Medicine and Respiratory Diseases Co-constructed by Henan Province and Ministry of Education of P.R. China, Henan University of Chinese Medicine, Zhengzhou, China

**Keywords:** biomarkers, chronic obstructive pulmonary disease, lung-gut axis, multi-omics, precision traditional Chinese medicine, systems biology, traditional Chinese medicine

**Author** Kun Yang was erroneously assigned as **Co-corresponding author**. The correct **Corresponding Author** is Hailong Zhang.

**Affiliation 1** Department of Respiratory Diseases, The First Affiliated Hospital of Henan University of Chinese Medicine, Zhengzhou, China was erroneously given as 1 Department of Respiratory Medicine, The First Affiliated Hospital of Henan University of Chinese Medicine, Zhengzhou, Henan, China.

**Affiliation 2** The First Clinical Medical School, Henan University of Chinese Medicine, Zhengzhou, China was erroneously given as 2 The First Clinical Medical College of Henan University of Chinese Medicine, Zhengzhou, Henan, China.

**Affiliation 3** Collaborative Innovation Center for Chinese Medicine and Respiratory Diseases Co-constructed by Henan Province and Ministry of Education of P.R. China, Henan University of Chinese Medicine, Zhengzhou, China was erroneously given as 3 Provincial-Ministerial Jointly-Built Collaborative Innovation Center for Traditional Chinese Medicine Prevention and Treatment of Respiratory Diseases / Henan Provincial Key Laboratory of Traditional Chinese Medicine Prevention and Treatment of Respiratory Diseases, Henan University of Chinese Medicine, Zhengzhou, Henan, China.

The original article has been updated.

